# Biological Activity and NMR-Fingerprinting of Balkan Endemic Species *Stachys thracica* Davidov

**DOI:** 10.3390/metabo12030251

**Published:** 2022-03-16

**Authors:** Desislava I. Mantovska, Miroslava K. Zhiponova, Milen I. Georgiev, Kalina Alipieva, Ivanka Tsacheva, Svetlana Simova, Zhenya P. Yordanova

**Affiliations:** 1 Department of Plant Physiology, Faculty of Biology, Sofia University “St. Kliment Ohridski”, 8 Dragan Tsankov Blvd., 1164 Sofia, Bulgaria; d_mantovska@biofac.uni-sofia.bg (D.I.M.); zhiponova@biofac.uni-sofia.bg (M.K.Z.); 2 Laboratory of Metabolomics, Institute of Microbiology, Bulgarian Academy of Sciences, 139 Ruski Blvd., 4000 Plovdiv, Bulgaria; milengeorgiev@gbg.bg; 3 Institute of Organic Chemistry with Centre of Phytochemistry, Bulgarian Academy of Sciences, bl. 9 Acad. Georgi Bonchev Str., 1113 Sofia, Bulgaria; kalina.alipieva@orgchm.bas.bg (K.A.); svetlana.simova@orgchm.bas.bg (S.S.); 4 Department of Biochemistry, Faculty of Biology, Sofia University “St. Kliment Ohridski”, 8 Dragan Tsankov Blvd., 1164 Sofia, Bulgaria; itsacheva@biofac.uni-sofia.bg

**Keywords:** Lamiaceae, woundwort, in vitro cultivation, phenylethanoid glycosides, antioxidant activity, anti-inflammatory activity

## Abstract

*Stachys thracica* Davidov is a Balkan endemic species distributed in Bulgaria, Greece, and Turkey. In Bulgaria, it is classified as “rare” and is under the protection of the Bulgarian biodiversity law. The aim of our study was to develop an efficient protocol for ex situ conservation of *S. thracica* and to perform comparative NMR-based metabolite profiling and bioactivity assays of extracts from in situ grown, in vitro cultivated, and ex vitro acclimated plants. Micropropagation of *S. thracica* was achieved by in vitro cultivation of mono-nodal segments on basal MS medium. Ex vitro adaptation was accomplished in the experimental field with 83% survival while conserved genetic identity between in vitro and ex vitro plants as shown by the overall sequence-related amplified polymorphism marker patterns was established. Verbascoside, chlorogenic acid, and trigonelline appeared the main secondary metabolites in in situ, in vitro cultivated, and ex vitro acclimated *S. thracica*. High total phenolic and flavonoid content as well as antioxidant and radical scavenging activity were observed in in situ and ex vitro plants. Further, the anti-inflammatory activity of *S. thracica* was tested by hemolytic assay and a high inhibition of the complement system was observed. Initiated in vitro and ex vitro cultures offer an effective tool for the management and better exploitation of the *Stachys* secondary metabolism and the selection of lines with high content of bioactive molecules and nutraceuticals.

## 1. Introduction

*Stachys* L., or woundwort, is a subcosmopolitan genus of herbs and shrubs that comprises more than 300 species [1] and is considered as one of the largest genera among the Liliaceae family. The plants from the genus are distributed in temperate and tropical regions of the Mediterranean, Asia, America, and Southern Africa. In Bulgaria, 22 species of the genus *Stachys* are naturally distributed, as five of them are under the protection of the Bulgarian biodiversity law [2]. Since ancient times, species of this genus have been used in traditional medicine under the form of extracts, decoctions, and ointments for the treatment of stomach disorders, genital tumors, skin inflammations, sclerosis of the spleen, cough, and ulcers [3,4]. In recent years, several studies on different taxa of genus *Stachys* have demonstrated that woundworts exert various biological effects, such as antioxidant, radical scavenging, anti-inflammatory, antibacterial, analgesic, hepatoprotective, cytotoxic, antiproliferative, wound healing, anxiolytic, and antidepressant potential [1,4,5,6]. Overall, based on these studies, *Stachys* species are considered as a potential source of nutraceuticals and functional ingredients and hence good candidates for pharmacological application.

Phytochemical studies in *Stachys* species revealed the presence of iridoids [7], polyphenols, including flavonoids [8], tannins, phenolic acids [5], phenylethanoid glycosides [9], diterpenes and triterpene saponins in addition to essential oil as minor constituents [4].

Plants are an indispensable reservoir of natural antioxidants which may find various applications as functional ingredients such as components of food supplements and natural food additives, as well as cosmetics and pharmaceuticals [1]. Natural antioxidants may very effectively reduce the lipid peroxidation in biological membranes, therefore they could be useful food additives and inhibitors of reactive oxygen species in living cells. Recent studies summarized by Tomou et al. [1] reported the high antioxidant potential of *Stachys sp*. and confirmed the possibility of their use as natural antioxidants. The ethyl acetate fraction of tubers of *S. affinis* showed extremely high DPPH radical scavenging activity (IC_50_ 0.85 ± 0.04 μg mL^−1^), several folds higher than the standard α-tocopherol (IC_50_ 18.68 ± 0.51 μg mL^−1^), which was attributed to the abundance of phenolics and flavonoids [10]. Another species, *S. mucronate* also demonstrated strong anti-radical activity due to the high content of polyphenols [11].

The anti-inflammatory activity of several *Stachys* species (*S. inflata*, *S. chrysantha*, *S. candida*, *S. athorecalix*, *S. beckeana*, *S. anisochila*, *S. plumose*, *S. alpina*, *S. germanica*, *S. officinalis*, *S. recta*, *S. schtschegleevii*) has been mainly prescribed to the presence of iridoids such as aucubin, acetylharpagide, harpagide, harpagoside, and ajugoside [6,12], as well as phenylethanoid glycosides (verbascoside and betonyoside F) and flavonoids [13].

*Stachys thracica* Davidov (The Plant List) or Thracian woundwort is a Balkan endemic plant distributed in Bulgaria, Greece, and Turkey. In Bulgaria, it is classified as “rare” and is under the protection of the Bulgarian biodiversity law. Thracian woundwort has several distribution locations with small populations in the Strandja Mountain, Black Sea coast, and the region of Sofia [14]. There is no available data on ex situ conservation of *S. thracica* and the knowledge of its chemical composition is rather scarce. Phenylethanoid glycosides and the diterpene betolide were isolated and identified in Thracian woundwort [15]. The aim of our study was to develop an efficient protocol for ex situ conservation of *S. thracica* and to perform comparative NMR profiling and bioactivity assays of extracts from in situ wild-grown, in vitro cultivated, and ex vitro acclimated plants.

## 2. Results and Discussion

### 2.1. In Vitro Cultivation and Ex Vitro Acclimation of S. thracica

**Figure 1:**
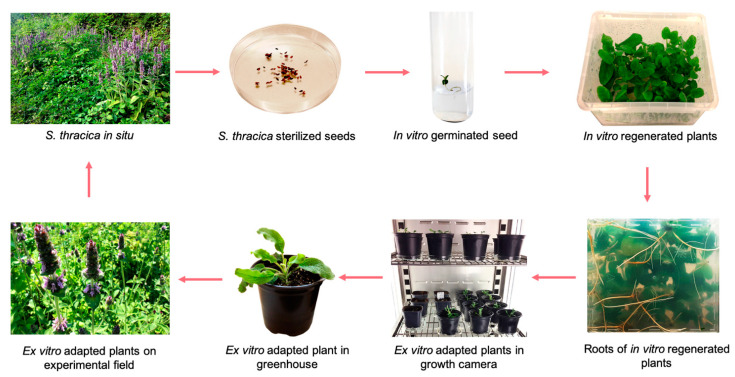
Ex situ conservation of *S. thracica*.

Bulgaria is characterized by a high level of endemism—approximately 270 species are considered Balkan endemics [16]. Most of those species have medicinal qualities and are a potential source of nutraceuticals and functional ingredients but as yet they have not been thoroughly studied. Micropropagation (in vitro cultivation) represents a biotechnological approach for ex situ conservation of rare and endangered plants, which can be carried out in a controlled environment regardless of seasons and climate changes [17]. Low seed germination of *S. thracica* seeds was found, as after 14 days only 20% of the seeds cultivated on 0.7% water agar germinated, while these inoculated on half-strength MS medium did not germinate. The sprouting seedlings were then transferred to a basal MS medium and cultivated under controlled environmental conditions. After one month, approximately, 78% of the explants showed shoot proliferation and produced 11.14 ± 0.73 shoots per explant. The regenerated micro-plants managed to form thick and healthy roots and plentiful leaf biomass (Figure 1).

The regenerated in vitro plants with 1 cm long shoots and a well-developed root system were transferred into pots with a sterile soil mixture. The first stage of acclimation was performed in a phytotron chamber under controlled environmental conditions and a gradual decrease in relative humidity. The survival rate of *S. thracica* acclimated plants appeared to be relatively high—83%. In the next step, the ex vitro adapted plants were transferred to a greenhouse and adapted for a period of one month followed by transfer to normal garden soil in the experimental field (Figure 1). The percentage of surviving plants remained unchanged.

Successful in vitro regeneration on hormone free MS medium and subsequent ex vitro adaptation has been previously reported for *S. maritima* [18]. However, in *S. leucoglossa* and *S. annua*, MS medium supplemented with 0.5 mg L^−1^ 6-benzyladenine was most effective for shoot proliferation and produced 5.61 ± 1.15 and 4.5 ± 0.54 shoots per explant respectively [19,20]. Root development in in vitro cultivated *S. leucoglossa* was established in 60% of the explants cultivated on WPM media supplemented with 0.5 mg L^−1^ indole-3-butyric acid [20].

### 2.2. Genetic Stability of In Vitro Cultivated and Ex Vitro Adapted S. thracica

The SRAP approach, developed by Li and Quiros [21], is a simple, effective, and reliable marker system that can be adapted for a variety of purposes. The SRAP markers have been successfully applied for analysis of genetic diversity in plant genetic resources and natural populations from the Lamiaceae family [22,23]. Alekseeva et al. [23] studied genetic diversity in *Origanum vulgare* subsp. *hirtum* and reported that the SRAP assay is sensitive in the detection of heterogeneity between individuals within populations of the same species. As well, comparison of SRAP profiles between different lavender varieties indicated high genetic diversity [22]. However, it has been pointed out that in the case of vegetatively propagated plants from one lavender variety, SRAP analysis with three primer pairs displayed the same pattern of SRAP fragments. In our work, a total of 16 combinations of SRAP primer pairs was selected for successful PCR amplification and generation of fragments for the polymorphism study of *S. thracica* plant variants. These primers showed sufficient efficiency to distinguish between *S. thracica* and other *Stachys* species (unpublished data). On the other hand, the comparison between in vitro and respective ex vitro *S. thracica* individuals demonstrated the presence of overall 496 different alleles without any variation in the SRAP profiles, which suggests preserved genetic stability during the process of micropropagation (Supplementary Table S1).

### 2.3. NMR-Based Metabolite Profiling during S. thracica Ex Situ Conservation

**Figure 2:**
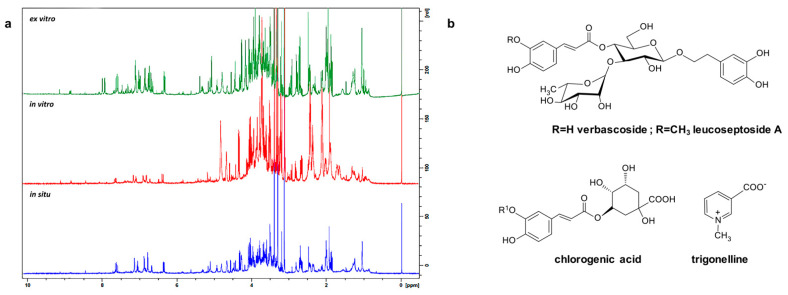
Metabolic profiling of *S. thracica* extracts: ^1^H NMR spectra of in situ, in vitro cultivated, and ex vitro adapted plants (**a**). Chemical structure of the main secondary metabolites identified in the extracts (**b**).

In order to reveal the metabolic alterations in *S. thracica* plants during the process of ex situ conservation an ^1^H NMR in combination with 2D NMR techniques (*J*-resolved, COSY, TOCSY, and HSQC) were applied. In total 15 individual compounds were unambiguously assigned to the abundant signals including carbohydrates, amino acids, organic acids, phenolic compounds, and alkaloids. No major qualitative differences were observed in the metabolic profiles of in situ, in vitro cultivated, and ex vitro adapted plants except for the two unidentified phenolic compounds, whose signals were detected only in the NMR spectra of ex vitro acclimated plants (Figure 2, Table 1). Verbascoside and leucoseptoside A along with chlorogenic acid and trigonelline appeared to be the main secondary metabolites in the extracts of all samples. Phenylethanoid glycosides are of common occurrence in the members of *Stachys* genus [4]. Verbascoside and leucoseptoside A have also been reported in *S. officinalis*, *S. recta*, *S. affinis*, *S. alpina* subsp. *dinarica*, *S. anisochila*, *S. beckeana*, *S. byzantine*, *S. plumose*, *S. iva*, *S. candida*, *S. schtschegleevii,* and others [1,4]. It has been found that verbascoside possesses a wide range of biological properties including antioxidant, anti-inflammatory, cytotoxic, antimicrobial, anti-thrombotic, and wound healing [24]. The identification of phenylethanoids was confirmed by comparison with authentic samples.

Chlorogenic acid is another major metabolite present with high intensity in the NMR spectra of all *S. thracica* samples. It is an important and biologically active dietary polyphenol found in the Asteraceae and Lamiaceae families [25]. Chlorogenic acid offers valuable therapeutic properties including antioxidant activity, antibacterial, hepatoprotective, cardioprotective, anti-inflammatory, antipyretic, neuroprotective, anti-obesity, antiviral, anti-microbial, anti-hypertension, and a central nervous system stimulation [25]. It has been also found that chlorogenic acid could modulate lipid metabolism and glucose in both genetic and healthy metabolic disorders [25].

The biological activities of trigonelline, a vitamin B6 derivative, have been thoroughly evaluated especially regarding diabetes and central nervous system disease. It possesses also hypoglycaemic, hypolipidemic, neuroprotective, antimigraine, sedative, and memory-improving activities, and may also reduce diabetic auditory neuropathy and platelet aggregation. It has been established that trigonelline affects β-cell regeneration, insulin secretion, activities of enzymes related to glucose metabolism, reactive oxygen species, axonal extension, and neuron excitability [26].

### 2.4. Comparative Determination of Total Phenols and Flavonoids in In Situ, In Vitro Cultivated, and Ex Vitro Adapted Plants

**Figure 3:**
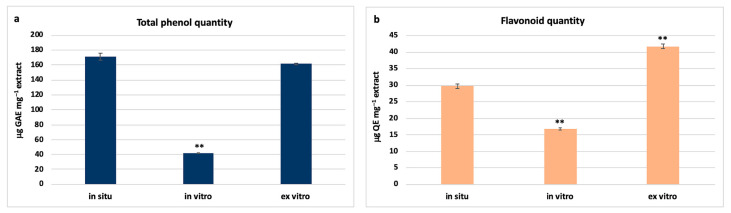
Total phenolic (**a**) and flavonoid (**b**) quantities in in situ, in vitro cultivated and ex vitro adapted *S. thracica* plants. Mean values ± SD are shown. Significant changes compared to in situ plants are indicated with asterisks **, (*p* ≤ 0.001).

Phenolic compounds are the most diverse and widely distributed secondary metabolites in plant species with pronounced antioxidant activity. They are synthesized mainly in the presence of adverse environmental factors (biotic and abiotic) and their amount varies significantly depending on the growth conditions. The determination of the total phenolic and flavonoid content in in situ, in vitro cultivated and ex vitro adapted *S. thracica* plants provides information on the quantitative changes in metabolic content in plants of the same genotype which are cultivated under different conditions. The highest phenolic content was established in in situ wild plants, followed by ex vitro adapted *S. thracica* (171 ± 4.5 and 161.6 ± 1.24 μg GAE mg^−1^ extract, respectively; Figure 3a). Reverse dependence was observed in the flavonoid content, which appeared highest in ex vitro acclimated plants followed by in situ grown (41.7 ± 0.68 and 29.7 ± 0.65 μg QE mg^−1^ extract, respectively; Figure 3b). A nearly fourfold decrease in the content of total phenols and a twofold decrease of flavonoids in in vitro cultivated *S. thracica* compared to wild growing genotypes was observed (Figure 3). It is likely that the aseptic culture conditions in controlled environment and mixotrophic nutrition affect the quantity of phenolic compounds in micropropagated *S. thracica* plants.

Strong inhibition of metabolic activity was also observed during in vitro cultivation of *Lamium album* and *Achillea thracica*, however, it recovered upon ex vitro adaptation [17,27]. In several plant model systems, a significant decrease in the concentration of total phenolic and flavonoid content was also found in in vitro cultivated plants compared to those grown in situ or adapted ex vitro [28,29].

### 2.5. Antioxidant and Radical Scavenging Activity of S. thracica during the Process of Ex Situ Conservation

**Figure 4:**
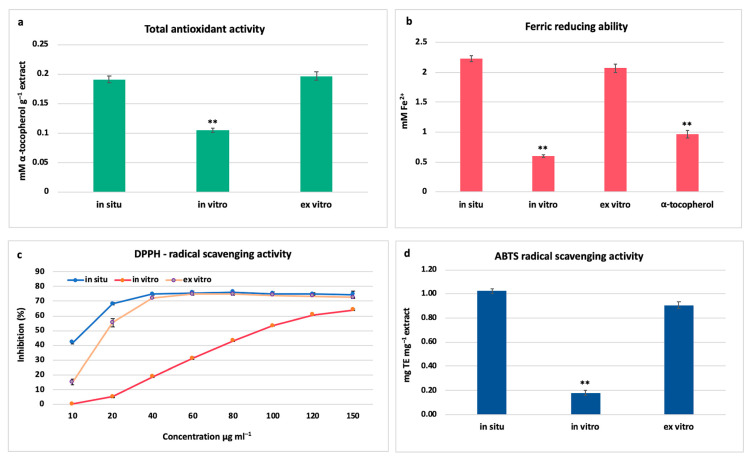
Antioxidant and radical scavenging potential of in situ, in vitro cultivated, and ex vitro adapted *S. thracica* plants: Total antioxidant activity (**a**). Ferric reducing ability of methanolic extracts compared to the standard sample of α-tocopherol (**b**). DPPH-radical scavenging activity (**c**). ABTS radical scavenging activity presented as Trolox equivalents (**d**). Mean values ± SD are shown. Significant changes compared to in situ plants are indicated with asterisks **, (*p* ≤ 0.001).

Phenolics have been recognized as essential antioxidant agents due to their structural characteristics and chemical behaviour. Based on the hydrogen-donating ability, they may act as free-radical scavengers and, consequently, exert a protective effect against reactive oxygen species (ROS) [30]. A positive correlation between the phenolic content and total antioxidant activity (TAA) was observed as the highest TAA was established in in situ and ex vitro acclimated plants (0.190 ± 0.005 and 0.197 ± 0.006 mM α-tocopherol g^−1^ extract, respectively). Approximately, two times lower TAA was found in in vitro cultivated *S. thracica* plants (0.104 ± 0.004 mM α-tocopherol g^−1^ extract; Figure 4a). Similar dependence was observed in the FRAP assay. The ferric reducing activity was highest in in situ wild and ex vitro adapted plants (2.23 ± 0.05 and 2.07 ± 0.07 mM Fe^2+^, respectively), and threefold lower in in vitro cultivated plants (0.598 ± 0.020 mM Fe^2+^; Figure 4b). In addition, the extracts from in situ and ex vitro acclimated plants have nearly two times higher antioxidant potential of the used standard α-tocopherol (Figure 4b). In DPPH assay all the three methanolic extracts exhibited concentration dependant radical scavenging activity. The maximum inhibition of the DPPH free radical was 76% and 74% at a concentration of 80 μg mL^−1^ for in situ and ex vitro adapted plants and 64% for in vitro cultivated *S. thracica* at the highest tested concentration—150 μg mL^−1^ (Figure 4c). The lowest concentrations at which 50% inhibition (IC_50_) of the DPPH radical was observed were 8.9 μg mL^−1^ for in situ, 24.4 μg mL^−1^ for ex vitro adapted, and 96.3 μg mL^−1^ for in vitro cultivated plants. The highest ABTS radical scavenging activity was detected in in situ wild and ex vitro adapted plants (1.02 ± 0.02 and 0.91 ± 0.03 mg TE mg^−1^ extract, respectively) and five-times lower activity was found in in vitro cultivated *S. thracica* (0.18 ± 0.02 mg TE mg^−1^ extract; Figure 4d).

Most likely the high antioxidant potential of in situ and ex vitro adapted *S. thracica* plants is due to the presence of verbascoside and chlorogenic acid, which are the main secondary metabolites found in the extracts through NMR profiling (Table 1). There is considerable evidence showing that phenylethanoids and phenolic acid are powerful antioxidants by scavenging ROS directly or acting as chain-breaking peroxyl radical scavengers [1,31]. It has been established that the major components, responsible for the high radical scavenging potential, in the methanolic extract of *S. officinalis* are verbascoside and chlorogenic acid, comprising 69% of the total antioxidant activity [31]. Methanolic extracts of aerial flowering parts of four *Stachys* taxa (*S. alpina* subsp. *dinarica*, *S. anisochila*, *S. beckeana* and *S. plumose*) were studied for their antioxidant activity and high correlations between total phenolic content, TAA and DPPH scavenging activity was established, indicating that polyphenols are the main antioxidants [12].

### 2.6. Anti-Inflammatory Activity of S. thracica

**Figure 5:**
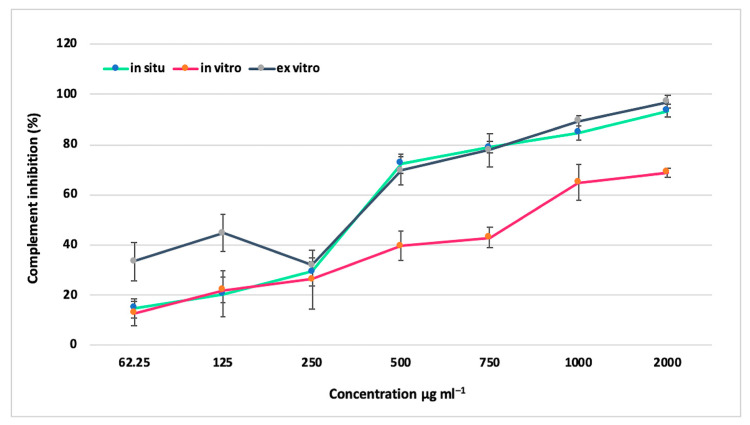
Inhibition of hemolysis by extracts of in situ, in vitro cultivated, and ex vitro adapted *S. thracica* plants. Mean values ± SD are shown.

The anti-inflammatory potential was examined by monitoring the effect of methanolic extracts of *S. thracica* on the complement hemolytic activity via the classical pathway (CP). The complement system is an innate effector mechanism with the intrinsic ability to initiate local inflammation at the sites of accumulation of ligands for its classical recognition molecule C1q, typically immune complexes, as well as to augment significantly an ongoing inflammatory response by the generation of plethora of pro-inflammatory mediators in a cascade mode. Therefore, inhibiting the complement activation (e.g., CP) would produce a profound anti-inflammatory effect. After treatment with the *S. thracica* extracts a dose-dependent inhibition on the CP was observed. The extracts from in situ and ex vitro adapted plants exhibited a similar degree of inhibition reaching up to 94% and 97%, respectively, at a concentration of 2000 μg mL^−1^, while those from in vitro cultivated plants reached a maximum of 69% (Figure 5). The lowest concentrations at which 50% inhibition (IC50) of the hemolysis was observed were 351.7 μg mL^−1^ for in situ, 358.5 μg mL^−1^ for ex vitro adapted, and 872 μg mL^−1^ for in vitro cultivated plants. The extracts from in situ and ex vitro acclimated plants had not only similar trends of inhibition on the CP but also a comparable anti-inflammatory effectiveness. The extract from in vitro cultivated *S. thracica* was 2.5-fold less active than the rest. It is very apparent that environmental factors significantly reduce the concentration of phenolic compounds and hence the biological activity of in vitro cultivated Thracian woundwort, while in ex vitro conditions, the biosynthetic capacity is restored, and this leads to an increase in bioactivity of the plants.

Maleki-Dizaji et al. [32] reported on the anti-inflammatory properties of a hydro alcoholic extract from *S. schtschegleevii* and associated this activity with the presence of phenylethanoid glycosides (verbascoside and betonyoside F) and flavonoids. It has been found that the polyphenol-rich extract from *S. officinalis* inhibited the enzymes lipoxygenase and cyclooxygenase-2 with IC_50_—1.22 μg mL^−1^ and 10.1 μg mL^−1^, respectively [33]. Haznagy-Radnai et al. [6] also reported high anti-inflammatory activity of aqueous extracts from *S. alpina*, *S. germanica*, *S. officinalis,* and *S. recta* in the carrageenan-induced paw oedema in rats. The extracts showed greater potency compared to the positive control diclofenac-Na.

## 3. Materials and Methods

### 3.1. Chemicals

Murashige & Skoog medium, plant agar, and sucrose used for in vitro cultivation were purchased from Duchefa Biochemie (Haarlem, The Netherlands). The organic solvents used for extraction, the reagents, and the standards used for the determination of total phenols and flavonoids, as well as the DPPH, ABTS, and FRAP free radicals were purchased from Sigma-Aldrich (Madrid, Spain). CD_3_OD and D_2_O came from Deutero GmbH (Kastellaun, Germany). The sensitized red sheep erythrocytes, hemolysin and the guinea pig serum were purchased from BulBio (Sofia, Bulgaria).

### 3.2. Plant Material and Culture Conditions

Wild grown in situ *Stachys thracica* Davidov plants were collected from their natural habitat (village of Sinemorets, Tsarevo municipality, Bulgaria) in the period of blooming in June and seeds in September, with the permission of the Ministry of Environment and Water of Bulgaria. A voucher specimen SO107847 was deposited in the Herbarium of Sofia University “St. Kliment Ohridski”. In Vitro shoot culture was induced by sterilization of 100 ripe dry seeds with 70% ethanol as described by Yordanova et al. [27]. After that, sterilized seeds were inoculated on half-strength Murashige and Skoog (MS) medium [34] and on 0.7% water agar (*w*/*v*). After 14 days the seedlings were transferred on MS medium with 3% (*w*/*v*) sucrose and 0.7% (*w*/*v*) agar and cultivated under controlled environmental conditions (80 μmol m^−2^s^−1^ photosynthetic active radiation, cool white fluorescent TL-D 36W/54-765 1SL/25 Philips, photoperiod 16 h light/8 h dark, 25 ± 1 °C, 50–60% moderate humidity). The plants were micropropagated twice over a period of 30 days before performing further experiments.

### 3.3. Ex Vitro Acclimation

In Vitro regenerated *S. thracica* plants with well-developed root systems were transferred to plastic pots containing a sterile soil mixture (peat:coconut-fibers:sand = 2:1:1). The first stage of ex vitro adaptation was performed for one month in a phytotron chamber (POL-EKO APARATURA SP.J.A. Polok—Kowalska KK 350 STD 1400 W) with 16/8 h light/dark, 100 µmol m^−2^ s^−1^ PPFD, 22 ± 2 °C, the relative humidity was decreased from 90% to 60% every week. In the next step, the ex vitro adapted plants were transferred to a greenhouse and adapted for a period of one month followed by transfer to normal garden soil at the experimental field of Sofia University “St. Kliment Ohridski”. After one year of acclimation to the field conditions, newly formed fully expanded leaves from the 2nd or 3rd nodes of the stem of ex vitro plants in the period of blooming were harvested and used for further analyses and NMR-metabolic profiling.

### 3.4. Genetic Stability Assay by SRAP Markers

To check genetic stability between in vitro and respective ex vitro adapted plants, the sequence-related amplified polymorphism (SRAP) approach was applied according to [21]. Frozen leaf material was used for genomic DNA purification and for subsequent PCR amplification, the primer pair sequences (ME1 to10 for forward primers targeting exons, and EM1 to 10 reverse primers targeting DNA non-coding region) used by Zagorcheva et al. [22] were utilized. In total, 16 SRAP primer pairs, which showed the highest number of peaks and clarity of the electropherogram, were selected for further use in the current study. The following primer pair combinations were used: ME1 + EM3; ME1 + EM5; ME1 + EM6; ME1 + EM7; ME1 + EM10; ME3 + EM2; ME3 + EM5; ME4 + EM5; ME6 + EM3; ME6 + EM5; ME7 + EM2; ME8 + EM1; ME8 + EM8; ME10 + EM4; ME10 + EM7; ME10 + EM9. The forward ME primers were 5′ end labelled with FAM (carboxyfluorescein) dye. PCR reactions were carried out with 50 ng genomic DNA, while the PCR amplification steps and fragment analysis were performed as described by [22]. The analysis of the length of the generated SRAP fragments was estimated by the GeneMapper Analysis Software v4.0 (Thermo Fisher Scientific Inc., Waltham, MA, USA). The threshold for peak detection was set to 400 relative fluorescence units. Fragment analysis was carried out for fragment sizes in the range of 50 to 950 bp.

### 3.5. Extraction Procedure and NMR Analyses

Extraction of the plant material and NMR analysis were described in detail by Zahmanov et al. [35]. Proton (^1^H) as well as 2D NMR spectra (*J*-resolved, COSY, HSQC, and TOCSY) were recorded at 25 °C on an AVII+ 600 spectrometer (Bruker, Karlsruhe, Germany), operating at a proton NMR frequency of 600.01 MHz [35]. Deuterated methanol was used for internal lock. The resulting ^1^H NMR spectra for each sample were phased, baseline corrected, and referenced to the residual signal of methanol-d_4_ at 3.30 ppm, by running TopSpin software (3.6.5, Bruker BioSpin Group).

### 3.6. Methanolic Extract Preparation

Three grams (3 g) of finely powdered dry plant material from aboveground parts of in situ grown, in vitro cultivated, and ex vitro adapted *S. thracica* was subjected to triple extraction with 30 mL chloroform (Sigma-Aldrich, Madrid, Spain) in an ultrasonic bath for 10 min. At the next step the biomass was extracted three times by distillation with methanol for 30 min. The final plant extract from each variant was concentrated through vacuum evaporator (IKA, Germany) and dried to constant dry weight. The yields of extracts from in situ, in vitro cultivated, and ex vitro adapted plants were 13.8%, 28.46%, and 13.6% respectively.

### 3.7. Determination of Total Phenolic and Flavonoid Content

The total phenolic content of *S. thracica* methanol extracts was determined using Folin–Ciocalteu reagent, according to the methodology proposed by Singleton et al. [36]. The determination of flavonoids was performed following the methodology of Chang et al. [37]. The content of polyphenols was quantified by a standard curve using gallic acid as a standard, and expressed as μg gallic acid equivalents per mg (μg GAE mg^−1^ extract). The flavonoid concentration was quantified by a standard curve using quercetin as a standard, and expressed as μg quercetin equivalents per mg extract (μg QE mg^−1^ extract).

### 3.8. Total Antioxidant Activity

Total antioxidant activity (TAA) was determined by the method of [38] with modifications. The incubation medium contains 0.25 mL extract (1 mg mL^−1^), 2.5 mL reagent (0.6 M H_2_SO_4_, 28 mM KH_2_PO_4_, and 4 mM (NH_4_)_2_MoO_4_). A control containing 0.25 mL of methanol and 2.5 mL of reagent was set in parallel and incubated under the same conditions. The samples were incubated in a water bath for 90 min at 95 °C. The reaction was quenched after placing the samples on ice and subsequent tempering to room temperature. Absorbance was measured at 695 nm on a spectrophotometer, Shimadzu 1800 UV. The TAA was expressed as mM α-tocopherol per gram extract (mM α-tocopherol g^−1^ extract).

### 3.9. DPPH Radical Scavenging Activity

The radical scavenging activity of methanolic extracts was determined using stable DPPH (1,1-diphenyl-2-picrylhydrazyl) free radical according to [39]. Eight different concentrations of the selected extracts were tested. Briefly, 0.5 mL of 0.1 mM DPPH solution, dissolved in methanol was added to 1.5 mL of sample. Methanol was used as a blank and a mixture of methanol (1.5 mL) with DPPH (0.5 mL) as a positive control. The samples were incubated for 30 min in the dark and the absorbance was measured at 517 nm. The results are given as maximum % inhibition and 50% inhibition (IC_50_) concentration in µg mL^−1^.

### 3.10. ABTS Radical Scavenging Activity

The ability of the different extracts to reduce the ABTS (2,20-azino-bis(3-ethylbenzothiazoline-6-sulphonic acid) free radical was determined by the method described by Re et al. [40]. The active ABTS free radical was produced by the reaction of 7 mM ABTS and 2.5 mM K_2_S_2_O_8_ in water. The solution is stored in the dark at room temperature for 12 to 16 h. The working ABTS solution was prepared by dilution of the active radical in methanol until the absorbance reached 0.7 ± 0.03 at 734 nm. An aliquot of 20 μL of each extract was mixed with 2.0 mL of the ABTS solution and after 1 min the absorbance was measured on a spectrophotometer, Shimadzu UV1800. Trolox was used as a standard. The results were expressed as mg Trolox equivalents per mg extract (mg TE mg^−1^ extract).

### 3.11. FRAP Assay

The ability of the extracts to reduce ferrous-containing radical was determined according to the method of [41]. The stock solution of the FRAP (Ferric Reducing Antioxidant Power) reagent was prepared by mixing 10 parts of 300 mM acetate buffer (pH = 3.6), 1 part of 10 mM TPTZ dissolved in 40 mM HCl, and 1 part of 20 mM FeCl_3_. The reaction mixture contained 1.8 mL of FRAP reagent, 0.2 mL of dH_2_O, and 0.040 mL of extract. Ten different concentrations of methanolic extracts were tested. After 30 min incubation at 37 °C the absorbance was measured at 593 nm on a Shimadzu UV1800 spectrophotometer. Ferric-reducing antioxidant capacity was represented as mM Fe^2+^ using an FeSO_4_ standard curve.

### 3.12. Microtitre Hemolytic Complement Assay

The hemolytic complement assay was performed in 96-well flat-bottom microtiter plates. The dried extracts were dissolved in 3% dimethyl sulfoxide (DMSO) and further diluted with barbitone buffered saline, pH 7.2, containing 0.15 mM Ca^2+^ (BBS). The assay was performed on 6% sheep erythrocyte (SE) suspension sensitized with rabbit polyclonal anti-SE serum (hemolysin, BulBio, Sofia, Bulgaria) and guinea pig complement (BulBio, Sofia, Bulgaria). Preliminary titration of sera was performed to determine the dilution producing 50% haemolysis of target erythrocytes. The SE (25 µL/well) were sensitized by 30 min incubation with hemolysin (dilution 1:1600, 25 µL/well) at 37 °C. After that complement (125 µL/well of appropriate dilution) and increasing amounts of the analyzed plant extracts (100 µL/well) were added and incubated for 1 h at 37 °C. Next, the microtiter plates were centrifuged at 1000× *g* for 5 min, and 200 µL of the supernatant from each well was transferred to new 96-well flat-bottom microtiter plates and the absorbance at 540 nm was measured by an ELISA reader (DR-200B, Hiwell Diatek Instruments, Jiangsu, China). Each assay was carried out in triplicate.

### 3.13. Data Analysis

The data are presented as mean ± SE of at least 12 scores (3 repetitions per variant in each of 4 independent sets of experiments). One-way ANOVA followed by Holm-Sidak statistical test with significance level 0.001 was performed with Sigma Plot 11.0 software to estimate the difference between all the variants.

## 4. Conclusions

The protocol for micropropagation of *S. thracica* comprises germination of sterilized ripe dry seeds on water agar and cultivation of sprouting seedlings on hormone free MS medium with 30 g L^−1^ sucrose for 30 days. Optimal induction of shoot proliferation from mono-nodal segments was achieved on MS medium without supplement of growth regulators. The ex vitro adaptation was accomplished on the experimental field and conserved genetic identity as shown by the overall sequence-related amplified polymorphism DNA marker patterns was established during the process of ex situ conservation. No significant changes in the NMR metabolic profiles, as measured by NMR, were found and verbascoside, leucoseptoside A, as well as chlorogenic acid and trigonelline were the most abundant metabolites in the extracts of *S. thracica*. As usual the aseptic conditions significantly reduced the quantity of total phenols and flavonoids and hence the antioxidant, radical scavenging, and anti-inflammatory potential of the extracts from in vitro cultivated plants. However, the biosynthetic potential and the associated biological activity were restored after adaptation of the plants to ex vitro conditions. The successful initiation of in vitro and ex vitro cultures is an alternative biotechnological approach for preservation of *S. thracica* which will also offer an effective tool for the management and better exploitation of the *Stachys* secondary metabolism and the selection of lines with high content of nutraceuticals and pharmaceutically valuable molecules.

## Figures and Tables

**Table 1:** Chemical shifts (*δ*) and coupling constants (*J*) of the metabolites, identified in *S. thracica* plants by the relevant 1D and 2D NMR spectra.

Metabolite	S.t. In Situ ^a^	S.t. In Vitro ^a^	S.t. Ex Vitro ^a^	Chemical Shift (ppm) ^b^
***Amino acids***				
Alanine	+	+	+	*δ* 1.47 (d, *J* = 7.2)
Valine	+	+	+	*δ* 0.99 (d, *J* = 7.0)/*δ* 1.04 (d, *J* = 7.0)
***Sugars***				
α-Glucose	+	+	+	*δ* 5.17 (d, *J =* 3.8)
β-Glucose	+	+	+	*δ* 4.56 (d, *J =* 7.9)/3.18 (dd, *J =* 7.9, 9.2 )
Sucrose	+	+	++	*δ* 5.37 (d, *J =* 3.8)
***Organic acids***				
Acetic acid	+	+	+	*δ* 1.92 (s)
Lactic acid	+	+	+	*δ* 1.31 (d, *J* = 6.9)/*δ* 4.08 m
Succinic acid	+	+	+	*δ* 2.48 (s)
Formic acid	+	+	+	*δ* 8.45 (s)
Malic acid	+	+	+	*δ* 2.80 (dd, *J* = 16.9, 8.2)/*δ* 2.93 (dd, *J*= 16.9, 3.9)
***Phenolic acids***				
Chlorogenic acid	++	++	+++	*δ* 7.60 (d, *J* = 15.7)/*δ* 7.13 (d, *J* = 2.2)/*δ* 7.06 (dd, *J* = 8.2, 2.2)/*δ* 6.86 (d, *J* = 8.3)/*δ* 6.33 (d, *J* = 15.9)/*δ* 5.30 (td, *J* = 4.9, 10.9)/*δ* 4.18 (br q, *J* = 3.1)
***Phenylethanoid glucosides***				
Verbascoside	++	++	+++	*δ* 7.63 (d, *J* = 15.9)/*δ* 7.14 (d, *J* = 2.0)/7.05 (dd, *J* = 8.3, 2.0)/*δ* 6.67 (dd, *J* = 8.3, 2.0)/*δ* 6.34 (d, *J* = 15.9)/4.93 (t, *J* = 9.6)/4.47 (d, *J* = 7.9)/*δ* 2.81 (t, *J* = 7.2) 1.04 (d, *J* = 6.4)
Leucosepthoside A	+	+	+	*δ* 7.70 (d, *J* = 15.8)/*δ* 7.23 (d, *J* = 1.9)/7.16 (dd, *J* = 8.3, 2.0)/*δ* 6.89 (dd, *J* = 8.3, 2.0)/*δ* 6.41 (d, *J* = 16.0)/4.93 (t, *J* = 9.6)/4.47 (d, *J* = 7.9)/*δ* 3.88 (s)/ *δ* 2.81 (t, *J* = 7.1) 1.04 (d, *J* = 6.4)
***Alkaloids***				
Trigonelline	+	+	+	*δ* 9.12 (s)/*δ* 8.83 (m)/*δ* 8.07 (m)/*δ* 4.43 (s)
***Others***				
Choline	+	+	+	*δ* 3.19 (s)
Unidentified phenolic compounds	-	-	++	*δ* 7.99 (d, *J* = 8.9)/*δ* 7.10 (d, *J* = 8.9) *δ* 7.93 (d, *J* = 8.9)/*δ* 6.99 (d, *J* = 8.9)

S. t. in situ—*S. thracica* in situ; S. t. in vitro—*S. thracica* in vitro; S. t. ex vitro—*S. thracica* ex vitro. ^a^ The sign “+” refers to relative fold differences and “–” to absence of the particular compound. ^b^ Proton NMR chemical shifts (*δ*) and coupling constant (*J*).

## Data Availability

Not applicable.

## Supplementary Materials

The following are available online at https://www.mdpi.com/article/10.3390/metabo12030251/s1, Table S1: Summary of SRAP alleles data following SRAP analysis of *S. thracica*.

## Sample Availability

Samples of the compounds are available from the authors.
