# Supplementary material for: Biological Activity and NMR-Fingerprinting of Balkan Endemic Species Stachys thracica Davidov
Source: Metabolites. 2022 Mar 16;12(3):251. doi: 10.3390/metabo12030251 (PMC8953131; doi:10.3390/metabo12030251)
Supplement: Supplementary file 1 [file metabolites-12-00251-s001.zip › metabolites-1626293-supplementary.pdf]

Supplementary Table S1

Table S1. Summary of SRAP alleles data following SRAP analysis of *S. thracica*.

**Primer Pair**	**SRAP Alleles for *S. thracica*[a]**
ME1_EM3	56
ME1_EM5	28
ME1_EM6	23
ME1_EM7	33
ME1_EM10	27
ME3_EM2	38
ME3_EM5	28
ME4_EM5	33
ME6_EM3	36
ME6_EM5	28
ME7_EM2	29
ME8_EM1	36
ME8_EM8	34
ME10_EM4	29
ME10_EM7	27
ME10_EM9	11
Total	496
